# The Immune Epitope Database (IEDB): 2024 update

**DOI:** 10.1093/nar/gkae1092

**Published:** 2024-11-18

**Authors:** Randi Vita, Nina Blazeska, Daniel Marrama, IEDB Curation Team Members, Deborah Shackelford, Leora Zalman, Gabriele Foos, Laura Zarebski, Kenneth Chan, Brian Reardon, Sidne Fitzpatrick, Matthew Busse, Sara Coleman, Caitlin Sedwick, Lindy Edwards, Catriona MacFarlane, Marcus Ennis, Sebastian Duesing, Jason Bennett, Jason Greenbaum, Marcus De Almeida Mendes, Jarjapu Mahita, Daniel K Wheeler, Jason R Cantrell, James A Overton, Darren A Natale, Alessandro Sette, Bjoern Peters

**Affiliations:** Center for Vaccine Innovation, La Jolla Institute for Immunology, La Jolla, CA 92037, USA; Center for Vaccine Innovation, La Jolla Institute for Immunology, La Jolla, CA 92037, USA; Center for Vaccine Innovation, La Jolla Institute for Immunology, La Jolla, CA 92037, USA; Center for Vaccine Innovation, La Jolla Institute for Immunology, La Jolla, CA 92037, USA; Center for Vaccine Innovation, La Jolla Institute for Immunology, La Jolla, CA 92037, USA; Center for Vaccine Innovation, La Jolla Institute for Immunology, La Jolla, CA 92037, USA; Center for Vaccine Innovation, La Jolla Institute for Immunology, La Jolla, CA 92037, USA; Center for Vaccine Innovation, La Jolla Institute for Immunology, La Jolla, CA 92037, USA; Leidos, Inc, San Diego, CA 92121, USA; Leidos, Inc, San Diego, CA 92121, USA; Knocean Inc, Toronto, Ontario M2P 2T3, Canada; Protein Information Resource, Georgetown University Medical Center, Washington, DC 20007, USA; Center for Vaccine Innovation, La Jolla Institute for Immunology, La Jolla, CA 92037, USA; Department of Medicine, University of California San Diego, La Jolla, CA 92093, USA; Center for Vaccine Innovation, La Jolla Institute for Immunology, La Jolla, CA 92037, USA; Department of Medicine, University of California San Diego, La Jolla, CA 92093, USA

## Abstract

Over the past 20 years, the Immune Epitope Database (IEDB, iedb.org) has established itself as the foremost resource for immune epitope data. The IEDB catalogs published epitopes and their contextual experimental data in a freely searchable public resource. The IEDB team manually curates data from the literature into a structured format and spans infectious, allergic, autoimmune, and transplant diseases. Here, we describe the enhancements made since our 2018 paper, capturing user-directed updates to the search interface, advanced data exports, increases in data quality, and improved interoperability across related resources. As we look forward to the next 20 years, we are confident in our ability to meet the needs of our users and to contribute to the broader field of data standardization.

## Graphical Abstract

**Graphical Abstract:**
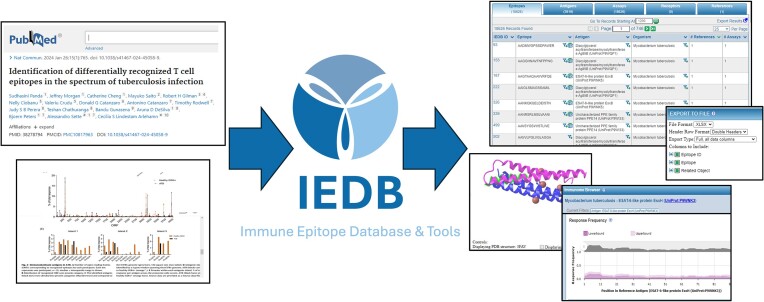

## Introduction

**Figure 1.**
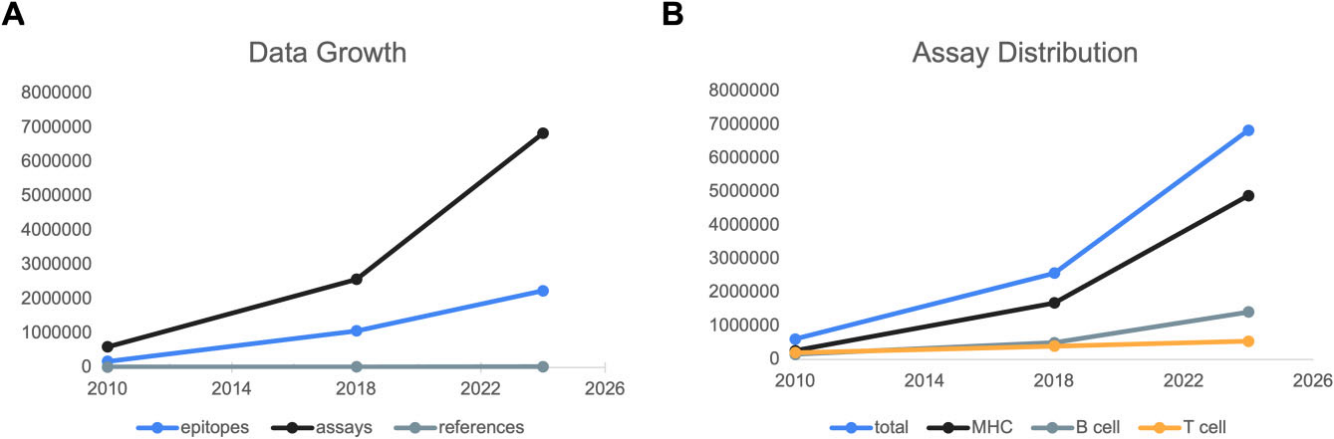
Data growth. Here, we present an overview of how the content of the IEDB has changed between the end of 2010, the first year that we were fully up to date with the literature, the end of 2018, when we last published a review on the IEDB, and current day. (**A**) The most growth has been noted in the numbers of assays per epitope and per publication. (**B**) MHC ligand assays have increased more rapidly than either B cell or T cell assays, as MHC ligand elution publications tend to elute thousands of epitopes per a single publication.

The Immune Epitope Database (IEDB, iedb.org) was established in 2004 as a free resource to house experimental data related to adaptive immune epitopes and to also provide leading-edge epitope analysis and prediction tools (1,2)). Over the past 20 years, the IEDB has extracted antibody, T cell, and major histocompatibility complex (MHC) experimental data from >25 000 publications, amounting to 6.8 million assays and 1.6 million immune epitopes. The scope of the IEDB includes data related to infectious, allergic, autoimmune, and transplant diseases in vertebrate hosts. Linear and discontinuous peptidic epitopes, as well as non-peptidic epitopes, are included. The curation of the scientific literature is up to date from 1952 to the present day. A new query of PubMed is performed every two weeks to maintain this status. Our data reflects the literature and is dependent upon what topics and research scenarios are published. For example, at the start of the COVID pandemic, the IEDB only had data on SARS-CoV1 epitopes, however, now we have 4.6 times the number of publications on SARS-CoV2 compared to CoV1, as new data was rapidly published. Figure 1 explores how the data in the IEDB has changed over the years. The number of references curated in the IEDB has largely maintained stable per year. At the same time, the number of assays added per year has massively increased, which upon further examination is primarily driven by MHC ligand elution publications, which tend to identify thousands of epitopes, and which may each be eluted from multiple MHC molecules (Figure 1A). Accordingly, when the types of assays within the IEDB are broken down by category, the greatest growth is seen in MHC assays, when compared to B cell and T cell assays (Figure 1B).

Our curation strategies are transparent and have been previously described (3). We have also previously illustrated how to use the various search features of our query interface (4). As a National Institute of Allergy and Infectious Diseases (NIAID) funded public resource, our chief purpose is to serve our users. We perform outreach to our user communities to drive the website layout, search interface and reporting options. Each fall, we hold a multi-day user workshop to describe any new features and demonstrate in detail how to access and use the data held within the IEDB. With almost 45 000 unique site visits per month, the IEDB has established itself as the key resource for immune epitope data. In this report, we will describe recent enhancements made to our search interface incorporating user community-specific filter options and a completely revised approach to perform protein-based queries using UniProt (5) Reference Proteomes. We also performed a major redesign of the data export formats and field options, added new help features, and added a query Application Programming Interface (API). Additionally, we illustrate how the continued integration of ontologies into the IEDB improves the quality of our data and drives interoperability and collaborations across related resources.

## Search interface

### New filter options

**Figure 2.**
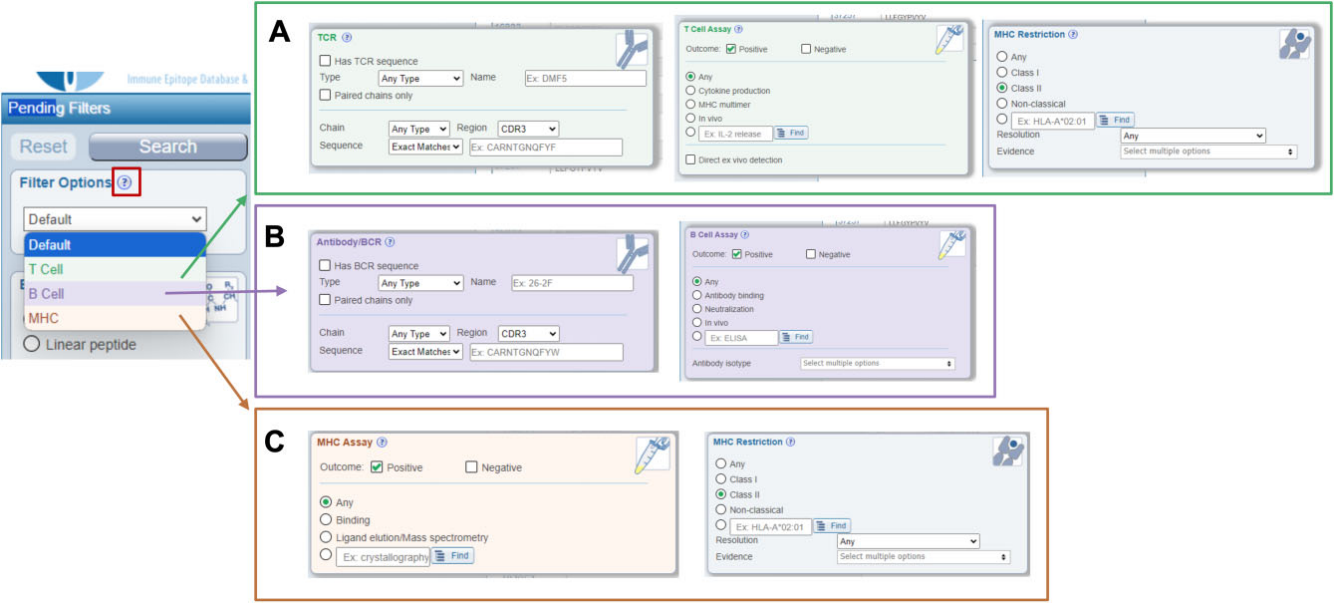
Filter options. These options convert the generic query interface into three specialized views with additional, community-relevant search parameters. The ‘?’ icon takes users to a short video and a detailed help article describing this new feature. (**A**) T cell options include parameters relevant to T cell researchers such as TCR, direct *ex vivo* analysis, and MHC details. (**B**) B cell filter options include antibody/BCR, isotype, and B cell specific assay types. (**C**) MHC options include MHC assay types and MHC molecule details such as resolution and evidence.

The IEDB serves multiple distinct user communities, including those studying antibody or T cell responses or MHC ligands. To meet the needs of individual communities, we added the concept of ‘Filter Options’, which provides users interested in one specific type of data with customized relevant search options (Figure 2). A short video and descriptive article describing this new feature can be accessed via the ‘?’ icon next to the ‘Filter Options’ header on the search interface. The ‘T cell’ Filter Option (Figure 2A) provides query features specific to T cell receptors (TCRs), such as the complementarity determining regions (CDR) 1, 2 or 3 or full length alpha or beta chain sequence, and is limited to T cell assay types, including cytokine production or MHC multimer experiments. Additionally, users can specify if the T cell response was studied *in vivo* or studied directly *ex vivo* - a concept highly relevant for T cell research with no obvious equivalent for antibodies. In contrast, the MHC restriction of T cell responses can be queried by MHC resolution and evidence. The resolution is a measure of how well-defined the author-provided MHC restriction is; at the class level (HLA class II), 4-digit allele for 1 chain (HLA-DPA1*01:03) or 4-digit alleles for both chains (HLA-DPA1*01:03/DPB1*02:01). The evidence is how the authors came to conclude the restriction; for example, they may have performed MHC binding prediction or experimental binding experiments. The ‘B cell’ Filter Option (Figure 2B) allows antibody researchers to query the features of the antibody, such as the isotype, heavy or light chain sequence, or the antibody name, and is limited to B cell assay types, such as neutralization assays. Features from the general search interface that are not relevant for antibody researchers, such as MHC restriction, are removed. The ‘MHC’ Filter Option (Figure 2C) narrows the data to either MHC binding or ligand elution/naturally processed assay types and, like the ‘T cell’ Filter Option, adds the ability to narrow data by MHC resolution and evidence. However, in this case, the MHC evidence is tailored to ligand elution experiments and includes scenarios such as antigen-presenting cells expressing a single allele or the use of MHC-specific antibodies.

### Protein tree updates

The vast majority (99.8%) of epitopes in the IEDB are peptides derived from naturally occurring proteins. Users frequently search for data based on the epitope's specific protein source. Thus, it is imperative that the ability to specify a protein of interest is intuitive and biologically meaningful. Data is added to the IEDB on a per-publication basis, where a curator enters all data from the publication as stated, which is then reviewed, validated, and promoted to the external website (3). This includes identifying the exact protein sequence of each peptide as described in the publication, typically via a Genbank (6) or UniProt accession. Thus, different publications studying the same epitope derived from the ‘same’ protein, such as SARS-Cov-2 spike protein, can be assigned to different sequence records (or isoforms) of the same protein. This may be due to natural sequence variability, or due to the availability of different protein sequences in external resources, such as UniProt, at the time each paper was curated. Regardless of the isoform selected, our typical users want to access all data from a given protein at one time.

**Figure 3.**
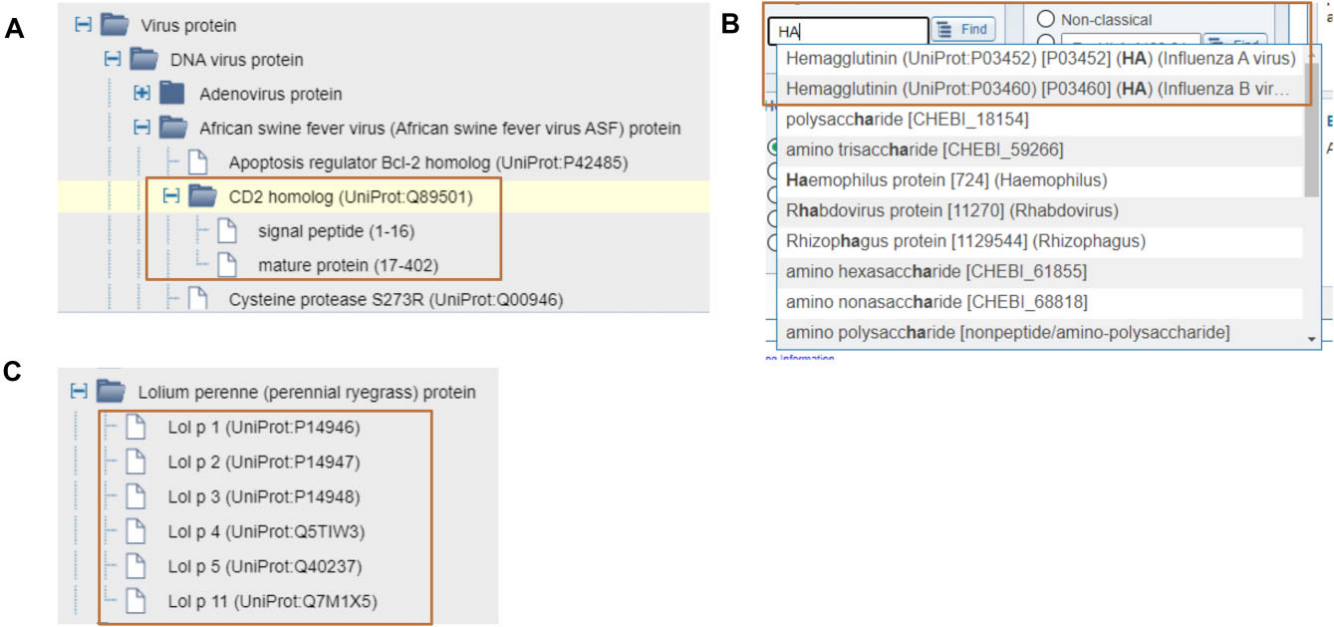
Protein tree. The Protein Tree allows us to present a vast amount of data in a simplified way. (**A**) Queries on specific fragments of a protein are enabled. (**B**) Synonyms imported from UniProt allow users to search by common names. (**C**) IUIS official nomenclature is used to present allergens.

To meet this need, we designed the ‘Protein Tree’, a hierarchical organization of protein sequence data that links each protein isoform to a ‘parent protein’. The development of the Protein Tree originally happened organically as an ever-growing set of scripts. This effort was completely restructured to take advantage of the massive increase in the availability of ‘Reference Proteomes’ in the UniProt database. Key steps in building the Protein Tree now include the identification of the best UniProt Reference Proteome for each species for which the IEDB has epitope data. Species are identified by the National Center for Biotechnology Information (NCBI) taxonomy, which is used as the basis for the main structure of the tree at the Organism level (7). The IEDB inserts custom nodes and synonyms to make the hierarchy more ‘immunologist friendly.’ Additionally, we prune out intermediate nodes, which are unnecessary. The curated IEDB data of epitopes and protein isoforms for each species are then mapped to a single entry in the chosen reference proteome (Figure 3). This Protein Tree is re-calculated with each weekly build to accommodate newly curated data and refreshed every 8 weeks to access newly available proteomes. By relying upon the UniProt Reference Proteomes, the Protein Tree gains additional capabilities. For example, UniProt captures how proteins are processed into fragments, such as signal peptides, which enables IEDB users to query on specific fragments of a protein (Figure 3A). UniProt also provides synonyms that are imported and allow our users to find their protein of interest by typing shorthand names (Figure 3B). Additionally, we incorporate custom features for immunologists. For example, we insert nodes to group MHC, B cell receptor (BCR), and TCR sources and we rely upon official International Union of Immunological Societies (IUIS) nomenclature for allergens as shown in Figure 3C (8). We also add synonyms when the terms commonly used by immunologists are not already present in UniProt. Our Solutions Center hosts articles describing the Organism and Protein Trees and is accessible via the ‘?’ icon next to the ‘Epitope Source’ header on the search interface.

## Data presentation and export

### Antigen summary page

**Figure 4.**
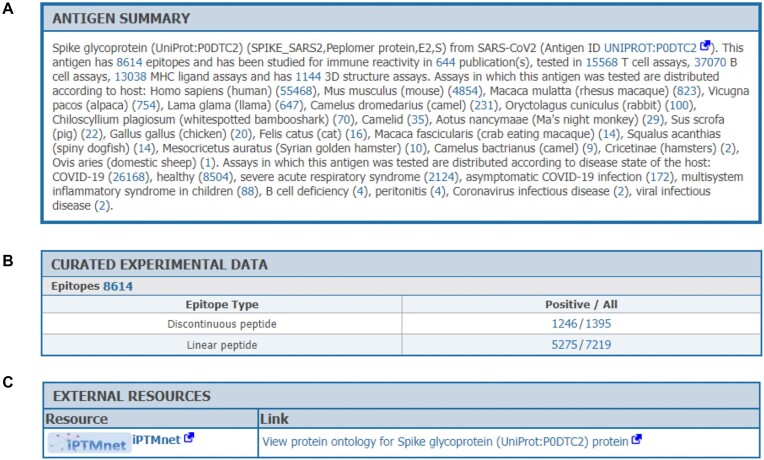
Antigen Summary page. This new page presents all information in the IEDB for any given antigen and also includes links to other resources. (**A**) The summary presents synonyms, counts of epitopes, publications, assays, 3D structures, and contextual information such as the hosts and disease states in which it was tested. (**B**) The compiled data for each protein, which is divided between linear and discontinuous epitopes, and shows how many of each had assays with positive outcomes versus how many assays were performed. (**C**) At the bottom of each Antigen Summary page, links out to other overlapping resources will be provided. We intend to add to this section over time, as new contacts are made.

A new feature in the IEDB is the Antigen Summary Page (Figure 4), which is accessed by clicking on the name of a protein on the Antigens tab of the Results page to display a summary of all data related to a given reference protein. The Summary, as shown in Figure 4A, includes synonyms, counts of epitopes, publications, assays, 3D structures, and contextual information such as the hosts and disease states in which it was tested. Figure 4B shows the compiled data for each protein separated into linear and discontinuous epitopes and shows how many of each had assays with positive outcomes versus how many assays were performed. Additionally, through a collaboration with UniProt, the Protein Ontology (PRO) (9) and IPTMnet (10), links from the IEDB to a variety of protein attributes in each of those resources are now made available to our users as shown in Figure 4C. PRO provides an ontological representation of proteins which groups proteoforms, isoforms, and orthologs in meaningful ways. IPTMnet catalogs the location and type of documented post-translational modifications in protein sequences. A help document on our solutions center describes the Antigen Summary Page in more detail.

### 3D viewers

**Figure 5.**
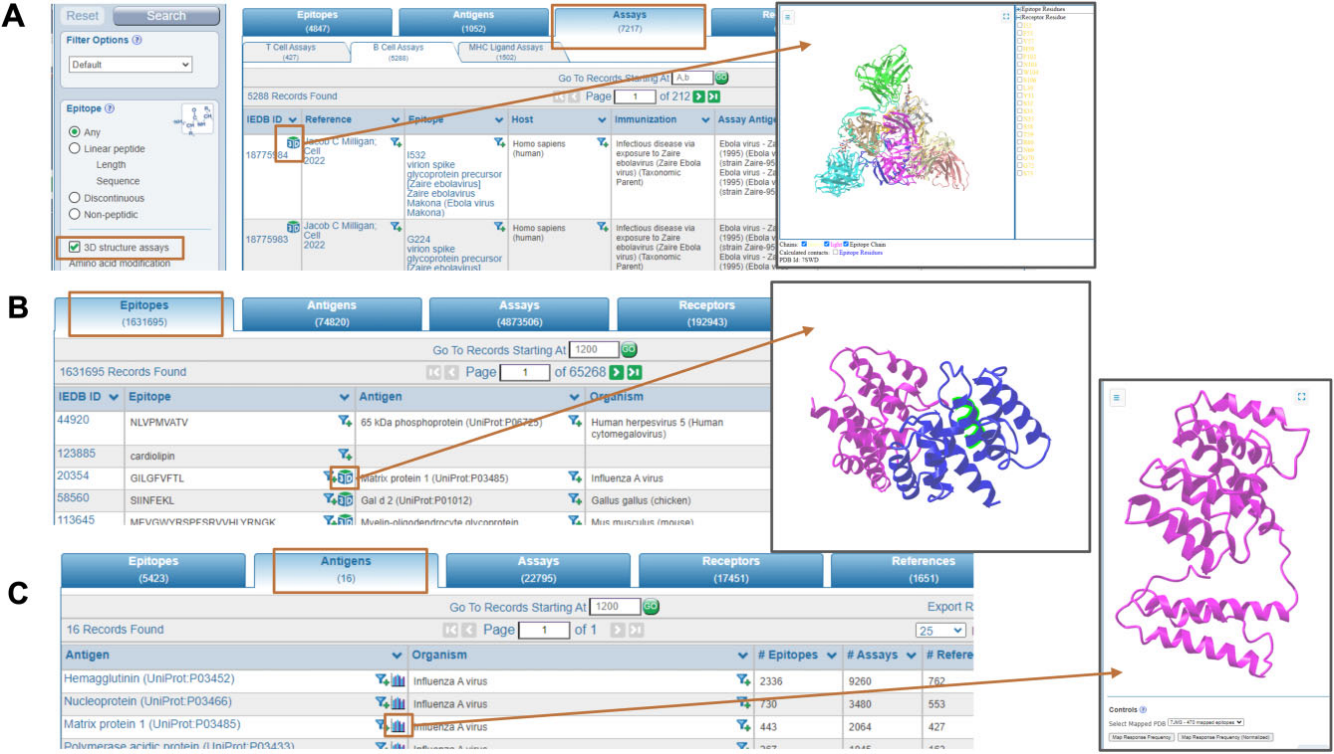
3D viewers. Three custom viewer applications are tailored for specific aspects of epitope visualization. (**A**) Experimentally-determined 3D structures of MHC-,TCR- or BCR-related complexes are accessible through the Assays tab. (**B**) Epitopes can be viewed in the context of the 3D structure or 3D model of the antigen through the Epitopes tab. (**C**) Immunogenicity regions on the antigen can be viewed through the ImmunomeBrowser tool.

Given the importance and interest in structural data, the IEDB updated its 3D viewer application (11). The IEDB 3D platform now utilizes NCBI’s iCn3D viewer (12) and offers three custom viewer applications tailored for specific aspects of epitope visualization. The Assay Viewer displays experimentally derived 3D structures from the Protein Data Bank (PDB) (13), illustrating an epitope in complex with MHC, TCR, or antibodies and the contacts between receptor and epitope (Figure 5A). The Epitope Viewer displays the location of individual epitopes on antigens using either experimentally derived 3D structures from the PDB or computationally derived 3D models from the AlphaFold Protein Structure Database (14) (Figure 5B). Lastly, the IEDB’s ImmunomeBrowser Viewer (15) maps multiple epitopes to an antigen, highlighting the immunogenic regions based on response frequency (Figure 5C). Together, the 3D viewers provide researchers with information on known structures, where available, and predicted antigen models for those without experimentally determined 3D structures using the AlphaFold Protein Structure Database. Each of these features has an article describing how to access and use it on our solutions center website, accessible via links at the bottom of each IEDB webpage and via ‘?’ icons when viewing the element.

### Data export

**Figure 6.**
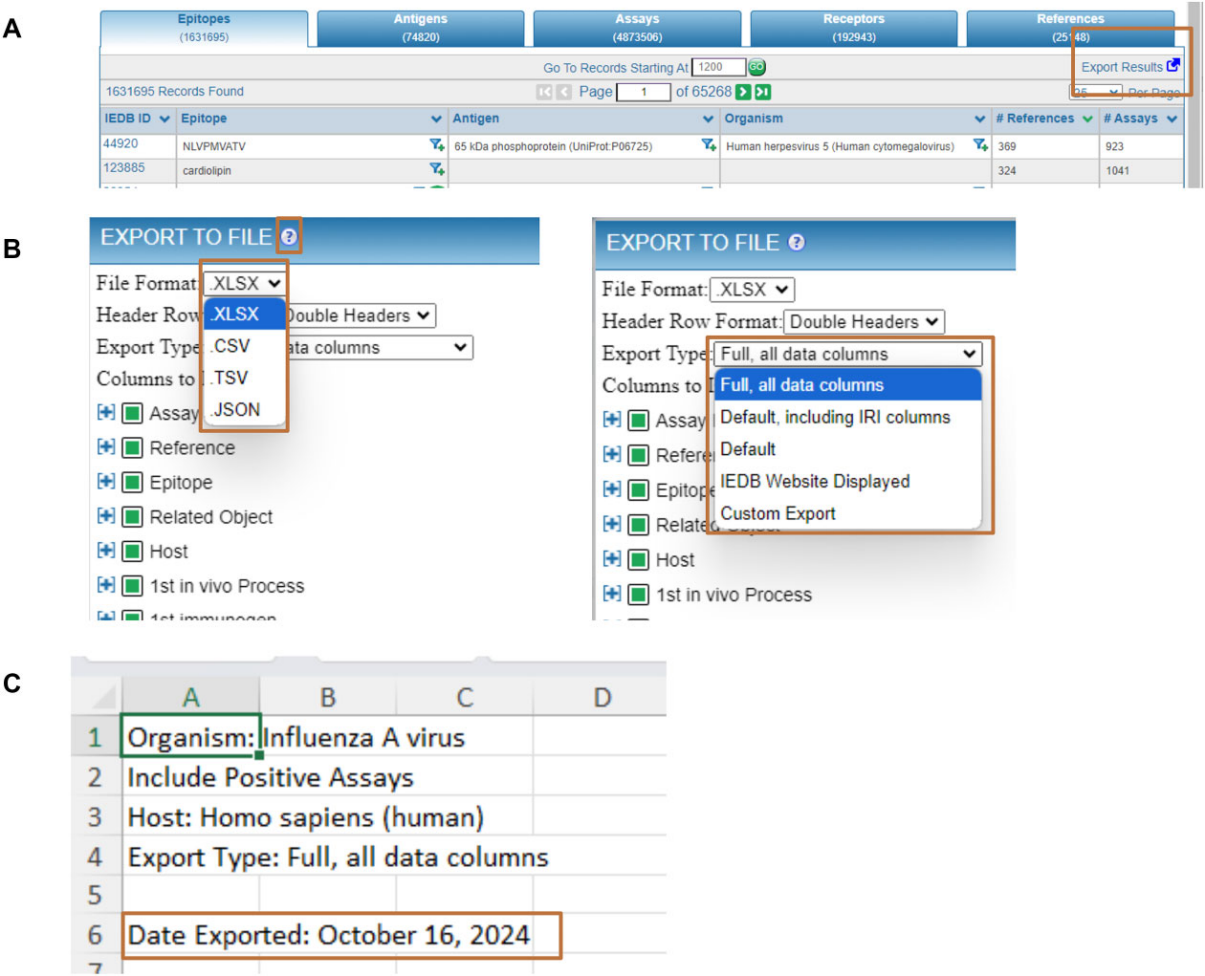
New export features. (**A**) Each tab of results can be exported via the ‘Export Results’ icon. (**B**) Exports are now available in a variety of file formats and users can select which columns of the data that they want to export. A ‘?’ icon takes users to a detailed help article describing the export features (**C**). Exports now include the query parameters and a help tab.

The purpose of the IEDB is to standardize and aggregate disparate data across many publications and to make this data available to the public. Due to the wealth of curated data, user queries often return very large relevant datasets that are not efficient to explore on a web interface. Thus, users must be able to download results in practical and meaningful formats for their own analysis. To improve upon our existing export capability, we redesigned the export options (Figure 6A). Based on user feedback, we implemented downloads in a variety of file formats (XLSX, CSV, TSV and JSON), included different options of how the ‘header’ information in the table is conveyed (single, double or no headers), and now allow the user to customize the columns included in the export or choose one of four commonly used preset types, all of which are described in more detail in a help article, accessible via the ‘?’ icon on the ‘Export to File’ header (Figure 6B). We also added two new elements to the XLSX exports. First, a ‘Help’ tab that provides a definition for each column of the export, as well as three example rows and a link to relevant help topics. Second, a ‘Query description’ tab that states the query that generated the results and a timestamp so users can better keep track of how and when their exports were generated.

### Application programming interface (API)

Based on user requests, the IEDB implemented a query API (IQ-API), to allow researchers programmatic access to the immunological data stored in the IEDB. Built on the PostgREST platform, it offers endpoints corresponding to various immunological entities, such as epitopes, antigens, assays (T cell, B cell and MHC), and receptor data. Users can perform detailed queries using PostgREST’s expressive syntax, with many filtering options, such as limiting data to specific pathogens or data associated with certain diseases, analogous to the IEDB frontend GUI. Users can receive results in JSON or TSV formats and use virtually any programming language to communicate with the API, with Python and R being the most popular choices. The API is free to use, and account registration is not required to make calls to it. This API can be used to integrate epitope data into bioinformatics workflows, supporting advanced research in immunology and vaccine development. Several use cases that illustrate some of the capabilities of the system are available at https://github.com/IEDB/IQ-API-use-cases using Python and R. Additionally, a help article describing all of the features of the API is available via the ‘More IEDB’ dropdown, located at the top of all IEDB webpages.

## Ontologies and interoperability

As an adopter of the FAIR principles (16,17), we continually integrate ontologies into the IEDB (18,19). We have a history of contributing to ontology development and establishing data standards in the immunology domain (20–23). In recent years, we reviewed the hundreds of fields needed to describe complex immune epitope assays with the aim of complete or nearly complete machine readability, semantic meaning, and therefore, seamless interoperability with overlapping resources employing the same strategies and ontologies. Adopting ontologies to standardize our data also improves the IEDB’s search functionality (24). Recently, we incorporated terms from the Vaccine Ontology (VO) (25) to describe adjuvants and a subset of human vaccines, Protein Modification Ontology (PSI-MOD) (26) to describe post-translational modifications, Evidence and Conclusion Ontology (ECO) (27) for evidence codes, and Ontology for Biomedical Investigations (OBI) (20), which was previously used for only assay types, for immunization routes. In each case, the process of mapping our unstandardized list values to a formal ontology allowed us to identify and correct errors in our dataset, and often we were able to add logic-based validation to prevent future errors. For example, certain post-translational modifications can only occur on specific amino acids. For our efforts to standardize the data in the IEDB to live up to their potential for interoperability, other relevant resources must also share in this same vision. Therefore, we seek out collaborations with related projects and contribute our strategies to external projects (28).

## Future plans

While the IEDB has matured as a resource, the field of immunology keeps on rapidly advancing, and so do the needs of our users. We aim to stay aware of user needs through outreach activities such as booths at national conferences, a help desk feature on the website, annual virtual user workshops, and by constantly seeking feedback from researchers, such as our expert committee. We will continue to enhance the existing features. For example, we are currently working on the addition of a gene layer to the previously described Protein Tree work, which will group reference proteome proteins by their gene, providing new insights into the origins of certain epitopes and adding the ability to search for epitopes by gene. We are also adding features to the Antigen Summary page, such as the gene information and new links to external resources gained through ongoing collaborations, such as with PRO.

To push our data standardization efforts even further, we are in the process of standardizing two of the remaining free text fields, ‘age’ and ‘data location’, currently available on the assay details pages and in the assay exports. The age field captures the age of the host at the time of the experiment and includes ages for any possible tested vertebrate species, for example, mice, humans, cows, etc. The data location field is used to specify where in the publication the curated experiment can be located, for example ‘Figure 1’, ‘Table IV’ or ‘Materials and Methods’. While standardizing these fields, errors are being identified and will be corrected. Methods to maintain standardization and prevent future errors will also be adopted.

We will continue to establish collaborations with overlapping projects and bring new features to our users. We hope that our successes at standardizing complex, contextual data will enable us to promote our methods and assist other resources move towards the same goal of true interoperability. To that end, we are currently collaborating with two wide-reaching external projects; the Integrated AIR Repertoire (AIRR) Knowledge Commons (i-AKC) [(airr-knowledge.org) and the Human Immunology Project Consortium (HIPC) Coordinating Center (HCC) (immunespace.org). The i-AKC project integrates existing, community-backed repositories containing data regarding adaptive immune receptors (AIRs) by bringing together and standardizing the disparate records available in VDJbase (29), Open Germline Receptor Database (OGRDB) (30), Immune Receptor Antigen Database (IRAD) (irad.roskinlab.org), and the IEDB. The HCC supports ImmuneSpace, a public web portal for human immune studies. This project increases the standardization of ImmPort (31) data and joins it with additional data from other overlapping repositories, such as the IEDB. As each of these projects mature, the data in the IEDB will become more interoperable and accessible through novel mechanisms, enabling new IEDB features.

## Data Availability

The Immune Epitope Database (IEDB) can be freely accessed at iedb.org.

## Appendix

### IEDB Curation Team Members:

Deborah Shackelford, Leora Zalman, Gabriele Foos, Laura Zarebski, Kenneth Chan, Brian Reardon, Sidne Fitzpatrick, Matthew Busse, Sara Coleman, Caitlin Sedwick, Lindy Edwards, Catriona MacFarlane, Marcus Ennis.
